# Global Perspective of Plant-Based Cosmetic Industry and Possible Contribution of Sri Lanka to the Development of Herbal Cosmetics

**DOI:** 10.1155/2022/9940548

**Published:** 2022-03-04

**Authors:** Dehel Gamage Nadeeshani Dilhara Gamage, Rathnayaka Mudiyanselage Dharmadasa, Don Chandana Abeysinghe, Rathnayaka Gamlathge Saman Wijesekara, Gamika A. Prathapasinghe, Takao Someya

**Affiliations:** ^1^Faculty of Agriculture and Plantation Management, Wayamba University of Sri Lanka, Makandura, Gonawila 60170, Sri Lanka; ^2^Industrial Technology Institute, 363 Bauddhaloka Mawatha, Colombo 7, Sri Lanka; ^3^Faculty of Livestock, Fisheries and Nutrition, Wayamba University of Sri Lanka, Makandura, Gonawila 60170, Sri Lanka; ^4^ALBION Co., Ltd, Ginza 1-7-10, Chuo-ku, Tokyo 104-0061, Japan

## Abstract

The global consumption of plant-based cosmetics has shown spectacular growth in recent years because of rising consumer awareness regarding the long-term health benefits of natural ingredients. As the global demand for herbal cosmetics increases, there are ample opportunities for Sri Lanka as a tropical Asian country to expand its productions and global exports along with its unique biodiversity and inherited traditional knowledge. Therefore, the present review attempts to give an overview of the widely used medicinal plants in the global herbal cosmetic industry and strengths, challenges, and possible solutions for the development of the herbal cosmetic industry of Sri Lanka. Information was collected using electronic search (using Pub Med, Science Direct, Web of Science, Google Scholar, TEEAL, and Scopus) for articles published in peer-reviewed journals, industrial reports, market surveys, and library search for local books on ethnobotany. Important plant-derived ingredients used in the global herbal cosmetic industry are essential oils, colorants, oils, fats, and waxes. The traditional usage of 108 medicinal plant species (belonging to 58 families) in cosmetic treatments was identified from the local books of Sri Lanka. Of these, 49 plant species were reported as new ingredients for the herbal cosmetic industry. However, the lack of ethnobotanical and ethnopharmacological surveys to identify the cosmetic potential plants, insufficient or absence of continuous supply of raw materials for production in line with the existing demand, the lack of quality control of raw materials and finished cosmetic products, improper systematic cultivation systems for medicinal plants, poor postharvest practices, and the lack of innovations are major challenges encountered in Sri Lanka for the development of the herbal cosmetic industry. In conclusion, addressing these vital knowledge gaps is a timely requirement of the country for the sustainable development of the herbal cosmetic industry in Sri Lanka. Furthermore, assembling of the multidisciplinary cooperation of botanists, chemists, toxicologists, researchers, and biologists is crucial to analyze the interesting functional properties, efficacy, and effectiveness of documented medicinal plants with cosmetic potential.

## 1. Introduction

In today's world, being presentable and looking great have become necessary for everyone [1]. The growing consumer demand for cosmetics is mainly driven by the aspiring young population who want to invest in grooming while maintaining their health [2]. Nonetheless, most individuals in different age groups use cosmetics in various forms [3]. With its huge profitability, it is a challenge for a manufacturer to provide a good quality product at a low cost that is simultaneously environmentally friendly [4]. Although cosmetic products are not generally associated with serious health risks, there are possibilities of adverse long-term effects of extensive usage of cosmetics [5]. For instance, cosmetics and toiletries may contain several hazardous ingredients: allergens, carcinogens, and endocrine disruptors. The various harmful allergens present in cosmetics may be grouped under phthalates, parabens, metals, chlorofluorocarbon propellants, and dioxanes. The bioaccumulation of these harmful chemicals and metals in the body over time has been associated with cancers, reproductive and developmental disorders, contact dermatitis, hair loss, lung damage, aging, allergies, and skin diseases [5, 6].

With the increasing awareness about the fewer side effects of herbal products [7], consumer demand for herbal cosmetics is becoming a rapidly growing segment globally [8]. The global natural and organic personal care products market is valued approximately at US$ 11 billion in 2016 and is expected to reach US$ 22 billion by 2022 [9]. North America is the major market for natural and organic personal care products, followed by Europe and Asia-Pacific. China and India are the specific countries that play a significant role in the global herbal cosmetics market. Natural and organic skin care maintains top billing in the global organic beauty market and is expected to emerge as the most attractive segment with a 30.9% share by 2024, followed by hair care [10].

Cosmeceuticals, which are cosmetic products containing naturally derived ingredients and fully organic cosmetic products, have become a trend at present. Consumer attraction for these products in the future is anticipated to grow significantly. Thus, the global demand for herbal cosmetics results in a huge trade from the local to the international level. At present, the majority of the developed countries' cosmetic manufacturers are continuously probing for new products and ingredients that are of tropical origin as their raw materials often have interesting properties because of varied climatic and topographical conditions [11]. Unfortunately, the plant-based cosmetic industry is still at its early stages in some tropical Asian countries, such as Sri Lanka, Vietnam, Indonesia, and Thailand. For the successful development of the herbal cosmetic industry in those countries, several challenges must be addressed. This review aims to give an overview of these points while providing widely used medicinal plants in the global herbal cosmetic industry. Furthermore, this review will highlight the possible contribution of Sri Lanka as a tropical Asian country to the development of herbal cosmetics.

## 2. Methodology

The process of bibliographic research was conducted from January 2020 to August 2020, comprehending works from 1999 to 2019. This review was mainly focused to address the following research questions:

1. What is the present status of the global herbal cosmetic industry, including market annual growth, leading cosmetic product categories, and the pioneers of the herbal cosmetic industry by geographical zones and countries?
2. What are the widely used specialty plant materials in global herbal cosmetic production and their functions?
3. What is the present status of the herbal cosmetic industry of Sri Lanka?
4. What are the strengths, challenges, and possible solutions for the development of the herbal cosmetic industry of Sri Lanka as a tropical Asian country?

The research was organized in two phases. Data extraction was done by two investigators (D.G.N.D. Gamage and R.M. Dharmadasa) independently at each phase. Any inconsistencies between the investigators were resolved by consensus with a third investigator (D.C. Abeysinghe). Information was gathered by adopting databases, such as Pub Med, Science Direct, Web of Science, Google Scholar, TEEAL, Wiley Online Library (Wiley), and Scopus.

During the first phase, information was collected on the global herbal cosmetic industry. Extensive bibliographic research was performed using keywords and syntax, such as “herbal cosmetics,” “global herbal cosmetic industry,” “medicinal plants with cosmetic potential,” “specialty plant materials used for cosmetic production and their functions,” “essential oils,” “plant-based dye and colorants,” “plant oils used in cosmetics,” “fat and waxes for cosmetics,” “herbal cosmetic industry in China,” “herbal cosmetic industry in India,” “medicinal plants used in cosmetics in China,” “Cosmetic treatments and traditional Chinese medicine,” and “medicinal plants used in skin care/hair care/oral care in India.” Furthermore, references listed in relevant journals were also screened. 218 journals were identified using databases, whereas 20 web resources, 13 industrial reports, 01 book, and 03 book chapters were identified using “Google” search. However, 93 journals, 14 web resources, 10 industrial reports, 01 book (International Cosmetic Ingredient Dictionary and Handbook, published by Personal Care Products Council, Washington), and 03 book chapters (The Therapeutic Benefits of Essential Oils, Chinese Topical Herbal Treatments and Essential Oil Protocols and Cosmetics' Quality Control) were utilized for writing on the global herbal cosmetic industry after excluding repeated records, suspected plagiarisms, non-peer reviewed journals, incomplete data or obvious errors of information (e.g., errors in scientific, vernacular, or English names of medicinal plants), and records in different languages. Materials in the English language alone were chosen during this phase. Furthermore, peer-reviewed journals and books, market analysis done by reputed research organizations (e.g., Future Market Insights/FMI), industry reports developed by governments or recognized non-governmental associations (e.g., Basic Chemicals, Cosmetics and Dyes Export Promotion Council, India/CHEMEXCIL, The Associated Chambers of Commerce and Industry of India/ASSOCHAM), ethnobotanical and ethnopharmacological surveys with minimum sample size and *in vitro* bioactivity studies of medicinal plants with a minimum number of replications were considered to be eligibility materials for writing in this phase. Moreover, extracted data from the publications on medicinal plants with cosmetic potential in China were compared with the “Inventory of Existing Cosmetic Ingredients in China (IECIC)-2015” issued by the National Medical Products Administration, China to confirm the usage of identified plant materials in the cosmetic industry. Cross-checking was performed by two investigators (D. G. N. D. Gamage and R. G. S. Wijesekara). However, no inventories or dictionaries of cosmetic ingredients were found for data comparison in India.

During the second phase, information on the herbal cosmetic industry of Sri Lanka, the traditional usage of medicinal plants in cosmetic treatments, strengths, and challenges for the development of the herbal cosmetic industry of Sri Lanka was accumulated. The bibliographic research was performed using keywords and syntax, such as “herbal cosmetics of Sri Lanka,” “herbal cosmetic industry of Sri Lanka,” “medicinal plants used for cosmetic treatments in Sri Lanka,” “medicinal plants and bioactivity studies in Sri Lanka,” and “medicinal plants used in skin care/hair care/oral care in Sri Lanka.” The numbers of records identified using databases and “Google” searching were 95 and 17, respectively. After applying the same inclusion and exclusion criteria, only 14 publications and 2 conference proceedings (Current scenario on the import of plant-based Ayurvedic raw materials in Sri Lanka and Current scenario of herbal medicine in Sri Lanka) were accepted for writing in this phase. Because of the lack of relevant publications available in databases, data extraction was focused by searching local books from the libraries of Industrial Technology Institute, National Science Foundation and Institute of Indigenous Medicine, University of Colombo in Sri Lanka. Therefore, both English and Sinhala languages were considered during the search of libraries. Four Ayurveda authentic books, namely the “Compendium of medicinal plants-Sri Lankan study, volumes I, II, III, and IV,” published by the Ayurveda Department of Sri Lanka and the book called “A collection of medicinal plants in Sri Lanka,” published by Nature's Beauty Creations Limited, Sri Lanka, were selected to identify potential medicinal plants for cosmetic productions. The current usage of identified medicinal plants with cosmetic potential through books was compared with the survey conducted on identifying medicinal plants used in the herbal cosmetic industry of Sri Lanka [12] to recognize new plant materials for the Sri Lankan herbal cosmetic industry.

**Figure 1:**
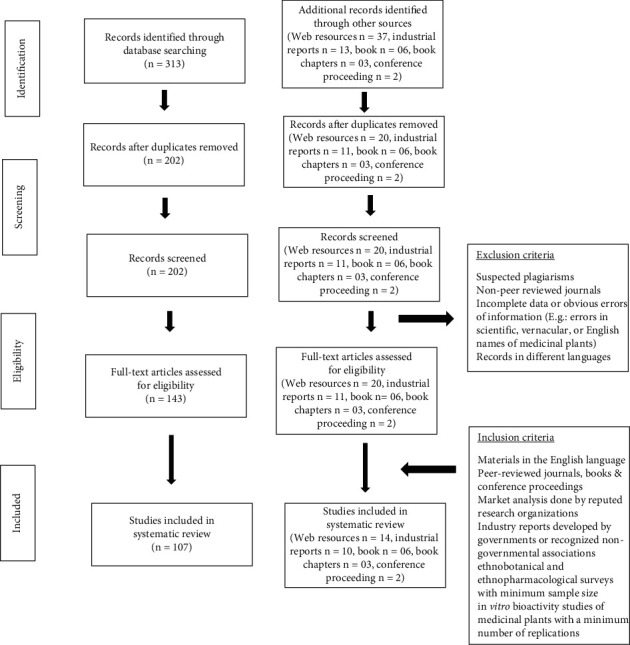
Flowchart of the study selection process.

In total, 107 peer-reviewed journal articles, 14 web resources, 10 industrial reports, 6 books, 2 conference papers from proceedings, and 3 book chapters were employed for writing this review.  Figure 1 illustrates the schematic diagram of the employed methodology. The results were summarized in a narrative manner using tables. The scientific names of documented plants were validated based on the collections listed on the homepages http://www.theplantlist.org and http://www.worldfloraonline.org. In addition, important links have been given where all details cannot be provided.

## 3. Results and Discussion

### 3.1. Global Perspective of the Plant-Based Cosmetic Industry

#### 3.1.1. Specialty Plant Materials for Herbal Cosmetic Production

In line with the U.S. Food and Drug Administration (FDA), the law defines cosmetics as “articles intended to be rubbed, poured, sprinkled, sprayed on, introduced into, or otherwise applied to the human body for cleansing, beautifying, promoting attractiveness, or altering the appearance” [3]. However, products that are formulated using various permissible cosmetic ingredients to form the base in which one or more herbal ingredients are used to provide defined cosmetic benefits, e.g., influencing the functions of the skin and providing nutrients necessary for healthy skin or healthy hair, can be described as “herbal cosmetics” [13, 14]. Free-radical scavenging, anti-inflammatory, antiaging, sun protection, the reduction of hyperpigmentation, and antimicrobial effects are some of the functional benefits [15]. In the formulation of herbal cosmetics, plants are used in three ways: a total extract, a single molecule obtained from the purification of extracts, or a selective extract [16]. Some important plant-derived ingredients used in cosmetics are oils, fats, waxes, essential oils, plant extracts, and colorants. These ingredients have numerous roles in the final cosmetic products, such as coloring, scenting, moisturizing, thickening, and stabilizing [15]. The usage amount of these specialty plant materials or their extracts in herbal cosmetic products is varied according to the product category, plant materials used, national, regional (e.g., EU cosmetics regulations), and international (e.g., The International Organization for Standardization (ISO), Food and Drug Administration (FDA)) rules and regulations. For instance, the allowable level of *Citrus bergamia* Risso (Bergamot oil) in cosmetics is 0.1 ppm in Austria. Furthermore, in line with the EU Cosmetics Regulation, the permissible concentration of essential oil in shower gels and baths (rinse-off products) is 0.01%, while the permissible concentration of essential oil in body oils, massage oils, and creams is 0.001% [17]. However, information on the acceptable concentrations of these individual specialty plant materials in cosmetics is scarce.

*(1) Essential Oils*. People have been using aromatic plants and oils for thousands of years in incense, perfumes, cosmetics, medicinal, and culinary applications [15]. Essential oils are highly concentrated, volatile, hydrophobic mixtures of chemicals extracted from plants. It is estimated that more than 3000 essential oils are of commercial importance and are used in flavor and cosmetic industries [18]. The largest consumer of essential oils is the United States of America, followed by western European countries, namely France, Germany, and the United Kingdom. At present, the United States and developing countries are dominant in the production of the most important essential oils. Essential oils are primarily used for their fragrance properties in cosmetic products. However, certain essential oils possess other interesting properties, such as antibacterial or antifungal, conditioning hair, and improving skin elasticity. Because of the unique functional properties and fragrances of plant-derived essential oils, the cosmetic industry uses them profoundly in a wide range of cosmetic products [11].  Table 1 lists the examples of plant-derived essential oils used in cosmetic products.

*(2) Dye and Colorants*. Currently, the market for natural colors in cosmetics continues to grow throughout the world. Manufacturers seek natural plant-based coloring materials that offer health benefits, such as antioxidant, antimicrobial, anti-inflammatory, antiaging, and UV protection properties, beyond their coloring properties alone [22–25]. At present, the main exporting countries of natural dyes are China, Peru, and India [11]. Plant colorants and pigments are used in a whole range of cosmetic products, such as creams, soaps, lotions, hair dyes, and make-up products [15, 26].  Table 2 lists various plant origin colorants and dyes used in the cosmetic industry.

*(3) Oils*. Oils are rich sources of fatty acids. Plant-derived oils from edible vegetables, fruits, seeds, plant seedlings, groundnuts, and trees have been safely consumed by humans for millennia. Various oils have been used on the skin since ancient times for cosmetic purposes. Oils are used as the base in a wide variety of cosmetic products, such as creams, emulsions, cosmetic milk, creams, ointments, hair conditioners, brilliantine, cosmetic masks, protective lipstick, bath fluids, nail varnish, and nail cleaners, along with their conditioning, occlusive, emollient, and moisturizing properties [28, 29]. As highlighted by Lubbe and Verpoorte [11], Zielinska and Nowak [29], and Athar and Nasir [30], Table 3 summarizes some commonly used plant-derived oils in cosmetic products.

*(4) Fat and Waxes*. Fats and waxes form an important group of ingredients for the manufacture of personal care products and decorative cosmetics. These are harder substances and very resistant to moisture, oxidation, and microbial attack. Waxes are widely used in a large variety of cosmetic products, such as creams, lotions, balms, ointments, lipsticks, mascara, foundations, and eye shadows for their emollient, moisturizing, thickening, and emulsifying properties. Kokum butter (*Garcinia indica* (Thouars) Choisy), Sal butter (*Shorea robusta* Gaertn.), Illipe butter (*Shorea stenoptera* Burck), avocado butter (*Persea americana* Mill.), cocoa butter (*Theobroma cacao* L.), carnauba wax (*Copernicia prunifera* (Mill.) H.E.Moore), candelilla wax (*Euphorbia* spp.), berry wax (*Rhus verniciflua* Stokes), sunflower wax (*Helianthus annuus* L.), and rice bran wax (*Oryza sativa* L.) are the most commonly used plant-derived fats and waxes used in cosmetic industry [11, 30, 55].

#### 3.1.2. China and India as Premier Exporters of Herbal Cosmetics

As CHEMEXCIL (Basic Chemicals, Cosmetics, and Dyes Export Promotion Council), set up by the Ministry of Commerce and Industry, Government of India, and Singh [56] highlight, China is the largest exporter of herbal cosmetics. Traditional Chinese medicine (TCM) has a deep history of herbal cosmetics production. There are plentiful instances where herbal skincare therapies have been popularly used in TCM. Most cosmetic products belong to the two categories called “antiaging products” and “moisturizer products.” Herbal drugs utilized in TCMs for medicinal purposes are used in cosmetics and personal care products. Therefore, the use of TCM compounds in the production of cosmetic products is very much derived from the medicinal and pharmaceutical applications of TCM [57]. TCM uses approximately 5000 plant species in a wide variety of herbal products [58].  Table 4 indicates some commonly used medicinal plants in TCM for cosmetic treatments [104].

However, *Angelica dahurica* Fisch.ex Hoffm., *Asarum sieboldii* Miq., *Asarum heterotropoides* F.Schmidt, *Astragalus propinquus* Schischkin, *Evodia ruticarpa* (A.Juss.) Hook.f. & Thomson, *Hippophae rhamnoides* L., *Saccharina japonica* (J.E.Areschoug) C.E.Lane, C.Mayes, Druehl & G.W.Saunders, *Lavandula angustifolia* Mill., *Ligusticum striatum* DC. (Benth. & Hook.fil.) Franch., *Matricaria reticulata* L., and *Pinus tabuliformis* Carrière, which were listed in Table 4, are no longer considered cosmetic ingredients according to the IECIC 2015.

IECIC 2015 was the latest version issued by the China Food and Drug Administration (CFDA) in 2015. It is a list of existing cosmetic ingredients that have already been used in cosmetics in China. According to IECIC [105], 8783 cosmetic ingredients are allowed to be used in China. Approximately, one-third of these ingredients are classified as “botanical extracts.” However, many of these ingredients are various formulations of the same plant material [104]. Cosmetic ingredients that are not listed are regarded as new cosmetic ingredients. Therefore, new cosmetic ingredients, including botanicals, must be approved by CFDA first before they can be used in cosmetics in China. Furthermore, more information about the approved cosmetic plant ingredients can be found from this link (http://www.cirs-reach.com/China_Chemical_Regulation/SFDA_Registration_of_New_Cosmetic_Ingredient_in_China.html).

In India, traditional medicine literature like the Ayurveda has proved the concept of using herbs for beautification in the past. The cosmetic preparations were used for worship and sensual enjoyment. Moreover, herbal extracts have been used for various skin and hair ailments and for enhancing the overall appearance over centuries. Over the last couple of decades, the Indian cosmetics industry has witnessed rapid and strong growth. Today, it is recognized as one of the emerging industries with immense growth potential [106]. Next to China, India is the largest producer of medicinal plants, and India owns more than 40% of global diversity [16]. According to the research report [107], India is one of the 12 mega biodiverse countries around the world. In India, nearly 45,000 plants are used in the Indian system of medicine, while 9,500 plant species are used by tribals in their daily requirements. Out of these 9500 species, 7,500 plants have direct medicinal use, while 950 are giving new leads and claims that require scientific scrutiny [108]. Furthermore, in India, more than 70% of the population uses herbal cosmetics for health care [109]. However, as indicated in the “International cosmetic ingredient dictionary and handbook” issued in 2016, India has not been included as the country that recognizes the need for uniformity in cosmetic ingredient nomenclature and has not formally identified dictionary (potential cosmetic ingredients) in its regulations [110].  Table 5 lists the most common herbal plants used for cosmetics and toiletries in India.

### 3.2. Sri Lanka as an Example for Tropical Asian Country: Possible Contribution to the Development of Herbal Cosmetics

Sri Lanka, formerly known as Ceylon, is an island with an area of approximately 65,610 km^2^. Despite its relatively small size, Sri Lanka possesses a high level of biodiversity because of its varied climate and topographical conditions. In view of that, it is recognized as a biodiversity hotspot of global and national importance. Sri Lanka has a traditional system of medicine, which is as ancient as the civilization of the island and practiced from generation to generation [116]. Natural resources, including herbal, mineral, and animal products, are the key resources of the Ayurveda and indigenous systems of medicine in Sri Lanka. Since time immemorial, plants have been playing a vital role in the healthcare system of Sri Lankans' livelihoods [117].

As specified in the literature, Sri Lanka has rich traditional systems of medicine, such as Ayurveda, Siddha, Unani, and Deshiya Chikitsa. It plays a pivotal role by fulfilling 60% to 70% of the rural populations' primary health care needs [118]. Sri Lanka is one of the most biologically diverse countries in Asia and currently possesses 29.7% of forest cover [119]. There are 3,771 flowering plant species, out of which about 927 (24%) are endemic to the country [120]. According to Sugathadasa et al. [121], 1,430 species representing 181 families and 838 genera can be considered medicinal plants. Out of the total number of species, 174 (12%) are endemic to Sri Lanka. About 250 species of medicinal plants are commonly used in traditional medicine, of which 50 species are heavily used [122]. The study conducted by Kankanamalage et al. [123] reveals the sources of medicinal plant materials that are obtained for numerous medicinal plant-based trades. Approximately 71.13% of these medicinal plants/herbal materials are obtained from local sources, and 26% are imported. Moreover, it reveals that 80% of the fresh and dry plant materials contribute to the herbal industry. Thus, it implies the importance of medicinal plants in different systems of medicine in Sri Lanka. Moreover, the study conducted by Dissanayake [124] on “Medicinal plant research in Sri Lanka: A scientometric study based on Scopus database” highlights the research studies of 190 plants, including 22 endemic plants. It reveals that most of the conducted studies were activity-based studies, such as toxicity, antibacterial, antifungal, hypoglycemic, antioxidant, anti-inflammatory, and diuretic activities. It was followed by general studies, such as physicochemical, chemical, postharvest, horticultural, and propagation studies of plants. This study shows the largely unexplored knowledge gap of medicinal plants in Sri Lanka.

However, considering the herbal cosmetics in Sri Lanka, the available data on herbal cosmetics' production and cosmetic potential medicinal plants are very scarce. Although the herbal cosmetic industry has exponentially increased throughout the world, the supply of potential cosmetic herbal products from Sri Lanka is still very limited. As Napagoda et al. [125] stated, only a handful of scientific evidence is available on the bioactivity studies of medicinal plants in Sri Lanka that could lead to the development of herbal cosmetics. Apart from the study on “cosmetic perspective of ethnobotany in the northern part of Sri Lanka,” [126] there has been hardly any ethnobotany report on the cosmetic potential of Sri Lankan medicinal plants. A total of 62 plant species belonging to 36 families have been identified based on the traditional knowledge and practices of the local community through this study. The identified plants are used for beautifying purposes, such as skin care, hair care, nail care, lip care, and eye care. However, the assembling of the multidisciplinary cooperation of botanists, chemists, toxicologists, researchers, and biologists is crucial to analyze interesting functional properties, efficacy, and effectiveness of documented cosmetic potential medicinal plants. Furthermore, Napagoda et al. [125] highlighted the probable usage of *Atalantia ceylanica* (Arn.) Oliv., *Hibiscus furcatus* Mullend., *Leucas zeylanica* (L.) W.T.Aiton, *Mollugo cerviana* (L.) Ser., *Olax zeylanica* L., and *Ophiorrhiza mungos* L. for the development of photoprotective cosmetic products by analyzing the antioxidant activity and the sun protection factor (SPF). Moreover, the research study conducted by Liyanaarachchi et al. [9] reveals the possible usage of *Artocarpus nobilis* Thwaites, *Artocarpus altilis* (Parkinson ex F.A.Zorn) Fosberg, *Elaeocarpus serratus* Heyne, *Curcuma aromatica*, and *Artocarpus heterophyllus* Lam. in the treatment of various skin disorders, such as hyperpigmentation, wrinkling, premature aging, and biological aging by analyzing tyrosinase, elastase, and hyaluronidase enzyme inhibitory and antioxidant activities.

Furthermore, Dissanayake [124] reports that most of the medicinal plant-related studies in Sri Lanka are still on the laboratory scale. Thus, investigating the cosmetic potential medicinal plants, isolation of active compounds, and bioactivity studies of medicinal plants has become necessary for Sri Lanka to support herbal cosmetic productions and innovations.

#### 3.2.1. Strengths of Sri Lanka as a Tropical Asian Country for the Development of Herbal Cosmetic Industry

Medicinal plants have played a pivotal role in many ancient traditional systems of medicine in Asia, such as the Ayurvedic and Unani systems of India and the traditional Chinese medicine and their derivatives in most Asian countries. Tropical Asian countries are unique among the geographical regions of the world because of high biological diversity, high cultural diversity, diverse ancient civilizations, and abundant raw materials. Because of their widely diversified ecological conditions, particularly in tropical rain forests, they have relatively high biological diversity that is the greatest of all world regions. This high diversity of the region is reflected by the number of species of plants and animals, including medicinal plants. The areas of high biological diversity are among the most culturally disparate, with large numbers of distinct communities inhabiting adjacent areas, each with its own language, culture, and system of traditional medicine. A wealth of traditional knowledge about medicinal plants to cure illnesses has been accumulated over a long period and has been handed down from generation to generation until the present time [127].

Medicinal plants have been used for centuries in medicinal, therapeutic, and beauty applications in Sri Lanka by different traditional systems of medicine, which have a documented history of over 2,500 years. Many formulae of medicinal preparations in the Sri Lankan traditional system of medicine are handed down from generation to generation. Some formulae are found only in the scripts of old “ola leaf” books treasured by traditional and Ayurvedic practitioners [128].  Table 6 lists the traditional usage of medicinal plants in Sri Lanka for cosmetic treatments.

As listed in Table 6, a total of 108 different plant species belonging to 58 families were identified from the books, “Compendium of medicinal plants, Sri Lankan study, Volume I to IV” and “A collection of medicinal plants in Sri Lanka.” The most dominant family was the family Fabaceae (13 plant species). A wide range of plant parts has been used for traditional cosmetic treatments. Based on the remedies, the identified plant parts were the leaves, bark, seeds, fruits, roots, flowers, rhizome, stem, heartwood, flower buds, tuber, gum, fruit rind, shoots, bulb, flower stamens, fruit kernel, inner bark, leaf gel, thorns, and wood. Medicinal plants are used in cosmetic treatments for various reasons, such as skin care, hair care, and oral care. *Myristica fragrans* Houtt. (Sadhikka) and *Kaempferia galanga* L. (Ingurupiyali) are reported in all three cosmetic treatment categories. Furthermore, 04 plants, namely *Ocimum tenuiflorum* L. (Heen Maduruthala), *Chrysopogon zizanioides* (L.) Roberty (Sawandara), *Citrus hystrix* DC. (Gada dehi), and *Curcuma zedoaria* (Christm.) Roscoe (Haran Kaha) are fragrant agents that have possible usage in the perfume industry.

The recent survey conducted by Gamage et al. [12] discloses the current usage of 115 plant species in herbal cosmetic productions in Sri Lanka. Furthermore, this study highlights the lack of proper cultivation systems of medicinal plants with cosmetic potential within the country for continuous herbal cosmetic productions. Although established cultivation systems are available for some highly used plants in the herbal cosmetic industry of Sri Lanka, such as *Aloe vera* (L.) Burm.f., most of the other medicinal plant materials are obtained either from the wild or through importation. Consequently, harvesting restrictions were imposed by the government for some wild species, such as *Coscinium fenestratum* (Goetgh.) Colebr. The adulteration of medicinal plant materials, lack of growers, declining suppliers, and lack of proper cultivation systems are major hindrances to the success of the herbal cosmetic industry in Sri Lanka. However, a comparison between the plant list identified through this survey and the plant list identified from the local books shows the possible usage of 49 plant species in the herbal cosmetic industry as new ingredients. Thus, these findings can be utilized for inventing new products in the future. As global demand for herbal cosmetics increases, there are ample opportunities for Sri Lanka to expand global export with its unique biodiversity and a rich base of traditional knowledge. It will lead to the upliftment of people's livelihoods and the county's economic development.

#### 3.2.2. Major Challenges and Possible Solutions for Sri Lanka to Develop Plant-Based Cosmetic Productions

At present, most developed countries' cosmetic manufacturers are continuously probing for new products and ingredients of tropical origin because their raw materials often have interesting properties. The interesting properties of tropical origin ingredients could vary based on climatic and topographical conditions [11]. However, the Association of Southeast Asian Nations (ASEAN) integration report issued in 2015 clearly indicates that developing cosmetic products and cosmetic product markets based on indigenous ingredients is one of the major challenges [133]. The lack of ethnobotanical and ethnopharmacological surveys to identify the cosmetic potential plants, insufficient or absence of continuous supply of raw materials for production in line with the existing demand, lack of quality control of raw materials and finished cosmetic products, improper systematic cultivation systems for medicinal plants, poor postharvest practices, lack of innovations, and the lack of efficacy tests (in vivo and in vitro) to ensure the safety are the major challenges found in Sri Lanka for the development of the herbal cosmetic industry [12, 104, 123, 124, 134].

To overcome the aforementioned challenges, collecting information on medicinal plants, traditional drugs, ethnobotany, and ethnopharmacology related to cosmetic treatments with traditional practitioners, Ayurveda physicians, and local communities in each country is required. As most of the traditional knowledge on medicinal plants and treatments in Sri Lanka is passed from generation to generation within families, conducting ethnobotanical and ethnopharmacological surveys will aid to identify cosmetic potential plants, important plant parts, remedies while preserving the traditional knowledge. Furthermore, information gathered through surveys could ultimately be utilized to develop the herbal cosmetic industry by the isolation and characterization of bioactive compounds from identified plants. Cosmetic companies work with a wide range of suppliers to obtain botanical raw materials. These suppliers purchase plant biomass from a wide array of sources ranging from large to small scale. However, harvesting plants from the wild is still popular for many medicinal herbs among local suppliers. Consequently, overharvesting can reduce plant populations to the point where the species' biodiversity is threatened, some species even to their extinction. Therefore, establishing proper systematic cultivation systems for identified medicinal plants is paramount. Furthermore, future research must be focused on nursery techniques, field establishments, and the harvest management of medicinal plants. Cultivated plant materials are preferred for the cosmetic industry as it is easier to control the whole supply chain and chemical variations. With the use of cultivated plants, problems such as adulteration and misidentification of material are mostly eliminated. It is also easier to adhere to quality standards and has less batch-to-batch variation as the plants are grown under controlled conditions. Furthermore, the utilization of biotechnology techniques, such as tissue culture, will benefit from preserving biodiversity by utilizing the endangered or unavailable plants using conventional production or wildcrafting. Product development and innovation are other key factors to succeed in the herbal cosmetic industry in Sri Lanka. The process to bring a medicinal plant from field to finished good can be quite complicated with many challenges along the way. Stability testing on raw materials to anticipate any issues with color change, odor, viscosity, precipitation, separation, or degradation of actives must be carried out routinely by cosmetic manufacturers. The characteristics of cosmetic products can be affected by environmental factors, such as temperature, pH, light, air, and humidity, which impact their stability contributing to severe damage to the constituents of the product [135]. Because of the wide variety of cosmetic products and their inherent complexity, it is hard to find standard stability tests that can be applied to a vast range of products. However, generally used stability tests can be categorized into several categories namely stability and physical integrity of cosmetic products (under appropriate conditions of storage, transport, and use), chemical stability, microbiological stability, and the compatibility between the contents and the container. For example, the organoleptic characteristics of cosmetic preparations, such as color, smell, texture, and consistency, can be evaluated by visual inspection. Additionally, several physicochemical analyses can be performed, such as centrifugation, mechanical vibration, light tests, pH, density, viscosity determination, and spectrophotometric assays, besides accelerated and microbial stability tests [136]. Quality control testing must be performed and continuously monitored to ensure its physical and analytical characteristics are up to standard. Some current techniques used by the cosmetic industry can be applied to the evaluation of cosmetic's quality control in an efficient manner, such as sensory analysis, rheology, and small-angle X-ray scattering [137]. Efficacy/safety tests should be performed on medicinal plant ingredients as well. For instance, cosmetics companies can perform in vitro testing to screen for cell damage in skin cell cultures or irritation in skin construct models and animal testing to avoid adverse events. However, the European Commission Cosmetics Directive has executed an animal testing ban on finished cosmetic products and ingredients. In such situations, cosmetic manufacturers have to find an alternative method to ensure product safety. At present, many alternatives to animal testing have been developed and validated for the safety and efficacy testing of cosmetic products and cosmetic ingredients. For example, 2D cell culture models derived from the human skin for evaluating anti-inflammatory properties, or predicting skin sensitization potential and 3D human skin equivalent models for evaluating skin irritation potential and excised human skin are being currently used as the gold standard for evaluating dermal absorption [138]. If a medicinal plant ingredient is safe and stable, it can move on to the formulation phase, where it is added to a cosmetic formula and retested for the same parameters, safety, and stability. With careful management, cosmetic companies can offer innovative beauty products that enhance living standards while conserving natural resources, promoting economic development for the poor, and protecting the environmental resources of Sri Lanka [11, 104, 108, 124, 127, 139, 140].

In addition, the cosmetics industry must employ scientists from the discovery stage to product development. Universities can play an active role in medicinal plant research in Sri Lanka. Universities can initiate transferring technology to industries for product development using active natural compounds. In the discovery stage, many different strategies should be used, including monitoring consumer trends, evaluating scientific advances in developed countries for possible technology transfer opportunities, monitoring scientific publications, and press reports. Furthermore, external collaborations with universities, institutes, or non-governmental organizations will secure the supply chains of raw materials, gain certification of their raw materials, or find higher quality and more sustainable plant-based raw materials. For instance, collaborative research studies on medicinal plants between different faculties in the Wayamba University of Sri Lanka, faculty of agriculture and plantation management, faculty of technology, faculty of medicine, and the pioneer semigovernmental research organization, Industrial Technology Institute in Sri Lanka would be ideal for new discoveries in herbal cosmetics discipline by sharing knowledge and utilizing available facilities to a maximum extent in these two entities. Through these partnerships, the companies not only increase cosmetic manufacturers' scientific knowledge and acquire innovative raw materials but also contribute positively to society [104, 124].

## 4. Conclusion

Medicinal plants provide accessible and culturally relevant sources of health care for most of the world's human population. There has been an increase in preference for herbal beauty products globally in recent years with rising consumer awareness regarding long-term health benefits. India and China have successfully utilized their traditional systems of medicine to become major producers in the global plant-based cosmetic industry. As cosmetic manufacturers in developed countries show their interest in herbal ingredients of tropical origin, Sri Lanka has a good potential to create numerous new avenues in herbal cosmetics, which can be easily capitalized on the global trends. However, the increasing interest in herbal cosmetics has raised important issues and highlighted vital gaps in the knowledge of cosmetics medicinal plants, their usages, cultivation technologies, postharvest technologies, and bioactivity studies in Sri Lanka. To overcome these issues, conducting ethnobotanical and ethnopharmacological surveys to identify medicinal plants with cosmetic potential, product development, and innovation in collaboration with universities, institutes, and non-governmental organizations are possible solutions. The study's findings could ultimately be utilized for the development of the herbal cosmetic industry by the isolation and characterization of bioactive compounds from identified plants while preserving traditional knowledge.

## Figures and Tables

**Table 1:** Examples for plant-derived essential oils used in cosmetic products.

Vernacular name	Scientific name	Functional property/properties	Reference(s)
Basil	*Ocimum basilicum* L.	Fragrance	[11]
		Antimicrobial properties	
Bay	*Laurus nobilis* L.	Fragrance	[11]
Bergamot oil	*Citrus bergamia* Risso	Fragrance	[19]
Calamus	*Acorus calamus* L.	Use in aromatherapy	[11]
Caraway	*Carum carvi* L.	Fragrance flavouring agent (mouth washes, toothpastes)	[11]
Cardamom	*Elettaria cardamomum* (L.) Maton	Use in aromatherapy	[11]
Carrot seed	*Daucus carota* L.	Use in aromatherapy	[11]
Citron	*Citrus medica* L.	Fragrance	[11]
		Antimicrobial properties	
Citronella oil	*Cymbopogon winterianus* Jowitt ex Bor	Fragrance	[11, 19]
Clary sage	*Salvia sclarea* L.	Fragrance	[11]
Garden sage	*Salvia officinalis* L.		
Spanish sage	*Salvia lavandulifolia* Vahl		
Clove	*Syzygium aromaticum* (L.) Merr. & L.M.Perry	Antimicrobial properties	[11, 20]
German chamomile	*Matricaria chamomilla* L.	Herbaceous odour	[17, 20]
		Anti-inflammatory and antiseptic properties	
Immortelle	*Helichrysum italicum* (Roth) G. Don	Fragrance	[17]
		Stimulates blood circulation in the skin and regenerates the skin	
		Antiwrinkle properties	
Jasmine oil	*Jasminum officinale* L.	Flowery fragrance	[19]
Lavender	*Lavandula angustifolia* Mill.	Sweet floral aroma	[11, 17, 20, 21]
		Anti-inflammatory, carminative, and sedative	
		Headache-relieving properties	
		Skin-healing properties	
Lemon	*Citrus limon* (L.) Osbeck	Antimicrobial properties	[11, 20]
Mint oil	*Mentha × piperita* L.	Fragrance	[11, 19, 20]
Neroli	*Citrus × aurantium* L.	Antimicrobial properties and antidepressant properties	[11, 17]
		Antiseptic properties	
		Carminative	
		Sedative properties	
Patchouli oil	*Pogostemon cablin* (Blanco) Benth.	Fragrance	[11, 19]
Rose oil	*Rosa × damascena* Herrm.		[17, 19]
		Antimicrobial, anti-inflammatory, and antioxidant properties, and moisturizing dry skin	
		Cleansing properties	
Rosemary oil	*Rosmarinus officinalis* L.	Fragrance (is widely used for hair care), nourishes the hair	[17, 19]
		Promotes hair growth	
		Antidandruff properties	
		Prevents hair loss	
Sandal oil	*Santalum album* L.	Fragrance	[11, 19]
Spike lavender	*Lavandula latifolia* Medik.	Antibacterial properties	
		Antiviral properties,	
		Anti-inflammatory, and nourishing properties	
Sri Lanka cinnamon	*Cinnamomum zeylanicum* Blume	Fragrance	[11]
		Antimicrobial properties	
Star anise	*Illicium verum* Hook.f.	Fragrance	[11]
Sweet orange	*Citrus sinensis* (L.) Osbeck	Antiseptic properties	[11, 20]
		Sedative	
		Carminative	
		Tonic	
Tea tree oil	*Melaleuca alternifolia* (Maiden & Betche) Cheel	Fragrance	[17, 20]
	*Melaleuca linariifolia* Sm.	Antiseptic properties	
	*Melaleuca dissitiflora* F.Muell.	Antifungal properties	
		Anti-inflammatory	
Vetiver oil	*Chrysopogon zizanioides* (L.) Roberty	Fragrance	[11, 19]

**Table 2:** Plants containing natural dye/colorant compounds used in the cosmetic industry.

English name	Source plant	Chemical class	Color	Reference(s)
Alkanet	*Alkanna* *tinctoria* (L.) Tausch	Alkannin	Red/purple	[24, 25]
Annatto	*Bixa* *orellana* L.	Norbixin, bixin	Orange/red	[11, 22, 27]
Butterfly pea	*Clitoria ternatea* L.	Delphinidin	Purple/blue	[24, 25]
Calendula	*Calendula officinalis* L.	Flavoxanthin	Orange	[24, 25]
Carrot	*Daucus carota* L.	Beta-carotene	Orange	[27]
Catechu	*Senegalia catechu* (L. f.) P.J.H. Hurter & Mabb.	Catechin	Red	[25]
Chamomile	*Matricaria recutita* L.	Chamazulene	Blue	[24, 25]
Dyer's woad	*Isatis tinctoria* L.	Alkaloid	Blue (indigo)	[11]
European barberry	*Berberis vulgaris* L.	Alkaloid	Yellow-brown	[11]
French marigold	*Tagetes patula* L.	Flavonoids	Yellow	[11, 22, 27]
Grape vine	*Vitis vinifera* L.	Anthocyanin	Red/blue	[11]
Henna	*Lawsonia inermis* L.	Naphthoquinone	Red	[11, 22, 27]
Hibiscus	*Hibiscus rosa-sinensis* L.	Cyanidin-3-sophoroside, cyanidin-3-sambubioside, delphinidin-3-sambubioside	Red/pink	[27]
Indigo	*Indigofera tinctoria* L.	Indigotin	Blue	[22]
Iris	*Iris* *×* *germanica* L.	Mangiferin, delphinidin	Purple/blue/green	[24, 25]
Madder	*Rubia tinctorum* L.	Alizarin, purpurin	Red/purple	[11, 27]
Paprika	*Capsicum annuum* L.	Carotenoids	Orange/red	[11, 22]
Persian walnut	*Juglans regia* L.	Naphthoquinone	Brown	[11]
Pomegranate	*Punica granatum* L.	Punicalagin	Red/purple	[22]
Red beet	*Beta vulgaris* L.	Betanin	Pink/red	[11]
Red cabbage	*Brassica oleracea* L.	Cyanidin-3-glucoside and delphinidin-3-glucoside	Pink/purple	[25]
Red sandalwood	*Pterocarpus santalinus* L.f.	Santalin	Red	[22]
Rosehip	*Rosa canina* L.	Lycopene, beta-carotene	Red/orange	
Safflower	*Carthamus tinctorius* L.	Flavonoid	Yellow/red	[11, 22]
Saffron	*Crocus sativus* L.	Crocin, crocetin, picrocrocin, riboflavin	Yellow	[22, 27]
Sappanwood	*Caesalpinia sappan* L.	Anthocyanin	Red	[24, 25]
Spinach	*Spinacia oleracea* L.	Chlorophyll	Green	[25]
Tomato	*Solanum lycopersicum* L.	Lycopene	Red/orange	[22]
Turmeric	*Curcuma longa* L.	Polyphenol	Bright yellow	[11, 22, 27]

**Table 3:** Some commonly used plant-derived oils in cosmetic products.

Oil	Source plant	Properties	Reference(s)
Almond oil	*Prunus dulcis* (Mill.) D.A.Webb	Reduces hypertrophic scarring, smoothing, rejuvenating, emollient, improving complexion and skin tone, anti-inflammatory, immunity-boosting	[31, 32]
Apricot kernel oil	*Prunus armeniaca* L.	Nourishing, revitalizing, emollient	[33]
Avocado oil	*Persea americana* Mill.	Hydrating, regenerating, antiaging, antiwrinkle, stimulating hair growth, having extraordinary transepidermal penetration capacity	[34]
Brazil nut oil	*Bertholletia excelsa* Bonpl.	Antioxidative	[35]
Camellia oil	*Camellia sinensis* (L.) Kuntze	Antimicrobial, antioxidative, antiallergic, antiviral, skin healing properties, antiwrinkle	[36]
Carrot oil	*Daucus carota* L.	Antiaging, antioxidant, suntan accelerator, photo protection, hair colour protection, emollient	[37]
Cashew nut oil	*Anacardium occidentale* L.	Antiaging, restoring moisture, smoothing	[38]
Castor oil	*Ricinus communis* L.	Emollient, lubricant, nourishing	[39, 40]
Coconut oil	*Cocos nucifera* L.	Emollient, hydrating, lubricating, cooling, soothing	[32]
Corn oil	*Zea mays* L.	Antioxidative, nourishing, antiaging, regenerating damaged cell membranes	
Cotton seed oil	*Gossypium hirsutum* L.	Emollient, cleansing, antioxidative, anti-inflammatory, soothing	[41]
Hyptis oil	*Hyptis suaveolens* (L.) Poit.	Emollient, antimicrobial	[42]
Jojoba oil	*Simmondsia chinensis* (Link) C.K. Schneid.	Soothing, healing, immune booster, antiacne, antibacterial	[32]
Linseed oil	*Linum usitatissimum* L.	Antiseptic, astringent	[43]
Marula oil	*Sclerocarya birrea* (A.Rich.) Hochst.	Moisturizing, occlusive	[44]
Neem oil	*Azadirachta indica* A.Juss.	Emollient, antiacne, immunostimulatory, antimicrobial, anti- inflammatory	[45]
Peanut oil	*Arachis hypogaea* L.	Emollient, anti-inflammatory	[32, 46]
Pine nut oil	*Pinus pinea* L.	Nourishing, curative, antiaging	[47]
Pumpkin seed oil	*Cucurbita pepo* L.	Antibacterial, antioxidative, anti-inflammatory	[48]
Rice bran oil	*Oryza sativa* L.	Antioxidative, antiaging	[49, 50]
Sesame oil	*Sesamum indicum* L.	Antioxidative, antiaging, healing effect	[32]
Soybean oil	*Glycine* max (L.) Merr.	Anti-inflammatory, skin lightening, antioxidative, antiaging, photoprotective	[51]
Starflower oil	*Borago officinalis* L.	Skin barrier repair effect, anti-inflammatory	[32]
Sunflower seed oil	*Helianthus annuus* L.	Emollient, moisturizing, nourishing, conditioning, antioxidative, antimicrobial	[32]
Walnut oil	*Juglans regia* L.	Antioxidative, moisturizing, antimicrobial, nourishing	[52, 53]
Watermelon seed oil	*Citrullus lanatus* (Thunb.) Matsum. & Nakai	Nourishing, soothing, skin lightening, moisturizing	[54]

**Table 4:** Some commonly used medicinal plants in TCM for cosmetic treatments.

Source plant	English name	Plant part	Function and usage	Reference/s
*Achyranthes* *bidentata* Blume	Ox knee	Root	Anti-inflammatory, antioxidant, antiaging properties	[59]
*Adenophora stricta* Miq.	Lady bell	Root	Humectant and skin conditioning	[60]
^*∗*^ *Angelica dahurica* (Hoffm.) Benth. & Hook.f. ex Franch. & Sav.	Chinese angelica	Root		
*Angelica sinensis* (Oliv.) Diels	Female ginseng	Root	Anti-inflammatory and antioxidant properties	[61]
^*∗*^ *Asarum* *sieboldii* Miq.	Chinese wild ginger	Whole plant		
^*∗*^ *Asarum* *heterotropoides* F.Schmidt	Chinese wild ginger	Whole plant		
^*∗*^ *Astragalus propinquus* Schischkin	Mongolian milkvetch	Root		
*Platycladus orientalis* (L.) Franco	Chinese arborvitae	Shoot	Anti-inflammatory properties	[62]
*Bletilla striata* (Thunb.) Rchb.f.	Chinese ground orchid	Rhizome	Antimicrobial, antioxidant, antiaging properties	[63]
*Bupleurum falcatum* L.	Chinese thoroughwax	Root	Anti-inflammatory and antioxidant properties	[64]
*Calendula officinalis* L.	Scotch marigold	Flower, leaves	Cleansing and antimicrobial properties	[65]
			Used in numerous cosmetic formulations, i.e., creams, lotions, shampoo	
*Camellia sinensis* (L.) Kuntze	Tea	Leaves	Anti-inflammatory, antimicrobial, antiviral, antioxidant properties	[66]
			Relieving skin damage and promoting wound healing	
*Centella asiatica* (L.) Urb.	Asiatic pennywort	Leaves	Wound healing, anti-inflammatory, antiviral, antibacterial, antifungal, and antioxidant properties	[67]
*Chrysanthemum indicum* L.	*Chrysanthemum*	Stem, flower	Anti-inflammatory properties	[68]
*Cimicifuga dahurica* (Turcz. ex Fisch. & C.A.Mey.) Maxim.	Silberkerze	Root	Anti-inflammatory, antiviral and antioxidant properties	[69]
*Coptis chinensis* Franch.	Goldthread	Rhizome	Antimicrobial properties	[70]
*Eucommia ulmoides* Oliv.	Eucommia	Bark	Antioxidant, anti-inflammatory, antimicrobial, and antiaging properties	[71]
^*∗*^ *Evodia* *ruticarpa* (A.Juss.) Hook.f. & Thomson	Evodia	Fruit		
*Forsythia suspensa* (Thunb.) Vahl	Weeping forsythia	Fruit	Anti-inflammatory, antioxidant, antiviral, antibacterial properties	[72]
*Ginkgo Biloba* L.	Maidenhair tree	Leaves	Antiaging properties	[73]
*Glycyrrhiza glabra* L.	European liquorice	Root	Antiaging, anti-inflammatory, and antioxidant properties	[74]
*Glycyrrhiza uralensis* Fisch.	Chinese liquorice	Root	Skin conditioning, antioxidant, and anti-inflammatory properties	[75, 76]
^*∗*^ *Hippophae rhamnoides* L.	Sea buckthorn	Fruit		
^*∗*^ *Saccharina japonica* (J.E.Areschoug) C.E.Lane, C.Mayes, Druehl & G.W.Saunders	Brown algae	Whole plant		
^*∗*^ *Lavandula angustifolia* Mill.	Common lavender	Flower, leaves		
*Lentinus edodes* (Berk.) Singer	Shiitake mushroom	Mushroom	Antimicrobial, anti-inflammatory, and antioxidant properties	[77]
			Faster skin renewal and increasing skin elasticity	
*Leonurus japonicus* Houtt.	Chinese motherwort	Fruit	Moisturizing, antiaging, and antioxidant properties	[78]
^*∗*^ *Ligusticum striatum* DC.	Szechwan lovage	Root		
*Ligusticum sinense* Oliv.	Chinese lovage root	Rhizome	Antimelanogenic and moisturizing properties	[79]
*Ligustrum lucidum* W.T. Aiton	Chinese privet	Root	Promoting growth and darkening of hair, reducing facial dark spots	[80]
*Lycium chinense* Mill.	Wolfberry	Fruit	Antiaging, anti-inflammatory, and antioxidant properties	[81]
*Magnolia biondii* Pamp.	Chinese willow leaves magnolia	Bark	Anti-inflammatory and antimicrobial properties	[82]
*Magnolia officinalis* Rehder & E.H.Wilson	Houpu magnolia	Flower	Antioxidant and anti-inflammatory properties	[83]
^*∗*^ *Matricaria reticulata* L.	Scented mayweed	Flower, seed		
*Morus alba* L.	Mulberry	Fruit	Antioxidant, anti-inflammatory, antimicrobial, and antiviral properties	[84]
			Tyrosinase inhibitors activity	
*Nelumbo nucifera* Gaertn.	*Lotus*	Leaves	Reduction of hyperpigmentation and antiwrinkling properties	[85]
*Paeonia × suffruticosa* Andrews	Peony	Leaves, root	Antioxidant, anti-inflammatory, and antiaging properties	[86]
*Panax ginseng* C.A. Mey.	Asian ginseng	Leaves	Antiaging and antiwrinkling properties	[87]
*Phellodendron amurense* Rupr.	Amur cork tree	Bark	Used to treat acne	[88]
^*∗*^ *Pinus tabuliformis* Carrière	Chinese red pine	Wood, leaves		
*Polygonatum officinale* All.	Solomon's seal	Root	Anti-inflammatory and healing properties	[89]
*Polygonum cuspidatum* Siebold & Zucc.	Japanese knotweed	Root	Potent tyrosinase inhibition, anti-inflammatory, antimicrobial, antiviral properties	[90]
*Polygonum multiflorum* Thunb.	Chinese knotweed	Leaves, root tuber, rhizomes	Tonic and antiaging agents	[91, 92]
			Used for promoting hair growth and treating early hair greying (blackening).	
*Prunus armeniaca* L.	Apricot	Seed	Antiaging, antioxidant, anti-inflammatory, antimicrobial, and radioprotective properties	[93, 94]
*Rehmannia glutinosa* (Gaertn.) DC.	Rehmannia	Root	Used to treat premature aging, greying hair, and wrinkles	[95]
*Rhodiola rosea* L.	Golden root	Rhizome	Antiaging and anti-inflammatory properties	[96]
*Salvia miltiorrhiza* Bunge	Chinese sage	Root	Anti-inflammatory and antioxidant properties	[97]
*Scutellaria baicalensis* Georgi	Chinese skullcap	Root	Antibacterial, antioxidation, and UV protection effects, and it can be used as a skin-whitening ingredient in the beauty industry because of its ability to inhibit melanin synthesis	[98]
*Silybum marianum* (L.) Gaertn.	Milk thistle	Seed	Antioxidant and UV B-protective properties	[99]
			Slowing down skin (photo) aging.	
*Sophora flavescens* Aiton	Korean cream pea	Root	Reduction of skin hyperpigmentation	[100]
*Tribulus terrestris* L.	Land caltrops	Fruit	Antiaging, anti-inflammatory, and antioxidant properties	[101]
*Vitis vinifera* L.	Grape	Fruit, seed	Antioxidant and skin conditioning properties	[102]
*Zanthoxylum alatum* Roxb.	Prickly ash	Bark	Antifungal, anti-inflammatory, and antioxidant properties	[103]
			Used for scouring teeth	

^*∗*^The plants that were not included in the “Inventory of Existing Cosmetic Ingredients in China (IECIC)-2015.”

**Table 5:** Most common herbal plants used for cosmetics and toiletries in India.

Category	Source plant	English name	Plant part	Function and usage	Reference(s)
Skin care	*Mangifera indica* L.	Mango	Plant	Antioxidant properties	[109, 111]
	*Juniperus communis* L.	Juniper	Whole plant	Rejuvenation properties	[109, 111]
	*Cuscuta reflexa* Roxb.	Dodder	Plant	Antimicrobial properties	[109, 111]
	*Phyllanthus emblica* L.	Indian gooseberry	Fruit	Antioxidant properties	[109, 111–113]
	*Withania somnifera* (L.) Dunal	Winter cherry	Whole plant	Antioxidant properties	[109, 111]
	*Cullen corylifolium* (L.) Medik.	*Psoralea*	Seeds	Antimicrobial properties	[109, 111]
	*Matricaria chamomilla* L.	Chamomile	Leaves	Antiacne properties, skin fairness properties	[109, 111]
	*Prunus dulcis* (Mill.) D.A.Webb	Almond	Kernel	Antiacne properties	[109, 113]
				Skin fairness properties	
	*Lagerstroemia speciosa* (L.) Pers.	Giant crape-myrtle	Leaves	Antiaging properties	[109]
	*Cydonia oblonga* Mill.	Quince	Seeds	Beautification and skin protection	[109, 111]
	Genus *Arctium*	Burdock	Root	Deep cleaning the pores and purifying the surface of the skin	[109]
	*Jasminum grandiflorum* L.	Spanish Jasmine	Flowers	Controlling skin diseases, protecting from sunburn	[109, 111]
	*Santalum album* L.	Sandalwood	Hardwood	Antioxidant properties and beautification	[109, 111]
	*Salvia hispanica* L.	Chia	Seeds	Antiaging properties	[109]
	*Euphorbia thymifolia* L.	Milk wort	Plant	Antimicrobial properties	[109, 111]
	*Cucumis sativus* L.	Cucumber	Peel	Cooling, toning, and skin-tightening properties	[109, 111]
	*Butea monosperma* (Lam.) Taub.	Bastard teak	Leaves and seeds	Antifungal properties	[109, 111]
	*Allium sativum* L.	Garlic	Cloves	Controlling sores, pimples, and acne	[109, 111]
	*Aloe vera* (L.) Burm.f.	Aloe	Leaves	Improving skin smoothness, healing, controlling skin burn	[109, 111]
	Genus *Vitis*	Grape	Seeds	Protecting skin elasticity	[109, 113]
	*Curcuma longa* L.	Turmeric	Rhizome	Anti-inflammatory and antioxidant properties	[109, 111–113]
	*Leucas aspera* (Willd.) Link	Thumbai	Leaves	Controlling scabies, skin psoriasis, chronic skin, skin eruption, and eczema	[109, 111]
	*Mallotus philippensis* (Lam.) Müll.Arg.	Monkey face tree	Flowers	Controlling scabies ringworm, leprous eruption	[109, 111]
	*Mimosa pudica* L.	Humble plant	Herb	Controlling itching	[109]
	*Rosa × damascena* Herrm.	Damask rose	Flowers	For beautification, smoothness, and protection from sunburns	[109, 111, 113]
	*Lavandula vera* DC.	Lavender	Inflorescence	Antiacne properties	[109, 111]
	*Lepidium meyenii* Walp.	Maca	Root	Promoting elasticity and maintaining the suppleness of the skin	[109]
	*Ailanthus excelsa* Roxb.	Tree of heaven	Leaves	Checking skin eruption	[109, 111]
	*Zea mays* L.	Maize	Stigma	Rejuvenation properties	[109, 111]
	*Cocos nucifera* L.	Coconut	Kernel	For skin itching and rashes	[109, 111, 113]
	*Azadirachta indica* A.Juss.	Neem	Bark, seeds, fruits and leaves	Antioxidant properties	[109, 111–113]
	*Citrus limon* (L.) Osbeck	Lemon	Fruit	Reducing skin itching and nourishing the skin	[109, 111]
	*Senna tora* (L.) Roxb.	Sickle senna	Leaves and seed	Antimicrobial properties	[109, 111]
	*Carica papaya* L.	Papaya	Leaves and seed	Improving skin softness and removing blemishes	[109, 111]
	*Plukenetia volubilis* L.	Mountain peanut		Promoting skin elasticity	[109]
	Genus *Hippophae*	Sea buckthorn		Nourishing the skin	[109]
	Genus *Helianthus*	Sunflower		Enhancing the brightness of the skin	[109, 113]
	*Sesamum indicum* L.	Sesame	Seeds	Rejuvenation properties	[109, 111]
	*Ocimum basilicum* L.	Basil	Leaves	Protecting from skin infections and rejuvenation properties	[109, 111]
	*Pistia stratiotes* L.	Water lettuce	Leaves	Controlling chronic skin disorders	[109, 111]
	*Justicia adhatoda* L.	Malabar nut	Leaves	Protecting skin and controlling scabies	[109, 111]
Hair care	*Juglans regia* L.	Walnut	Leaves and hull	Hair dyeing	[109, 113]
	*Aloe vera* (L.) Burm.f.	Aloe	Gel	Dissolving the dead skin cells and excessive sebum that can clog hair follicles	[109, 113]
	*Phyllanthus emblica* L.	Indian gooseberry	Fruit	Promoting hair growth	[109, 111, 114]
	*Ocimum basilicum* L.	Basil		Anti-inflammatory properties, strengthening hair against breakage, and improving circulation in the hair follicles, which helps to stimulate growth	[109]
	*Eclipta prostrata* (L.) L.	False daisy	Herb	Encouraging hair growth	[109, 111, 112]
	*Thymus serpyllum* L.	Wild thyme	Herb	Useful for preparing hair tonics	[109, 111]
	*Ficus racemosa* L.	Cluster fig	Aerial root	Checking falling hairs	[109, 111]
	*Terminalia bellirica* (Gaertn.) Roxb.	Belleric myrobalans	Seeds	Use for hair dyingpreparation	[109, 111]
	*Betula pendula* Roth	Birch	Leaves	Antidandruff properties	[109, 111]
	*Centella asiatica* (L.) Urb.	Pennywort	Whole plant	Improving circulation and promoting stronger hair growth	[109, 111, 114]
	*Terminalia chebula* Retz.	Ginger	Seeds	Use in hair care formulations	[109, 111]
	*Lawsonia inermis* L.	Henna	Leaves	Use for hair dyeing and nourishment	[109, 111–114]
	*Nardostachys jatamansi* (D.Don) DC.	Spikenard	Rhizome	Use in hair tonics for growth	[109, 111, 112, 114]
	*Lavandula angustifolia* Mill.	Lavender	Inflorescence	Stimulating circulation in the scalp, strengthening new hair growth, and helping balance the natural oil production of the scalp	[109]
	*Calendula officinalis* L.	Marigold	Flowers	Smoothening effect of hair	[109, 111]
	*Brassica* spp.	Mustard	Seeds	Nourishing the hair	[109, 111]
	*Cocos nucifera* L.	Coconut	Kernel	Use for preparing hair oils and tonics	[109, 111, 113, 114]
	*Mentha × piperita* L.	Peppermint		Use as a healing scalp treatment	[109]
	*Sapindus mukorossi* Gaertn.	Soap nut	Fruit coat	Natural shampoo and cleansing hair	[109, 111]
	*Carthamus tinctorius* L.	Safflower		Use in hair tonics	[109, 111]
	*Salvia officinalis* L.	Sage		Use as a hair conditioner	[109, 111]
	*Acacia concinna* (Willd.) DC.	Soap pod	Pods	Use as hair cleanser and for control of dandruff	[109, 111, 112, 114]
	*Sesamum indicum* L.	Sesame	Seeds	Major source of hair oils and use for preparing specific hair oils	[109, 111, 114]
	*Nasturtium officinale* R.Br.	Watercress		Supporting the stronger hair growth	[109]
Oral care	*Achyranthes aspera* L.	Prickly chaff flower	Root	Use as a toothbrush, good for dental caries	[115]
	*Argemone mexicana* L.	Mexican pricklypoppy	Seeds	Good for gum troubles	[115]
	*Azadirachta indica* A.Juss.	Neem	Twigs	Use to clean teeth and is considered good for dental caries and gum infection	[113, 115]
	*Berberis lycium* Royle	Indian lycium	Peeled stem	Considered good for scouring teeth	[115]
	*Calotropis procera* (Aiton) Dryand.	Sodom	Latex	Used for toothache	[115]
	*Capsicum annuum* L.	Paprika	Fruits	Good for toothache	[115]
	*Senna occidentalis* (L.) Link	Coffee senna	Leaves	Use for scouring teeth	[115]
	*Cinnamomum tamala (*Buch.-Ham.) T.Nees & Eberm.	Bay leaf	Leaves	Use for scouring teeth	[115]
				Good for gum inflammation	
	*Citrus limon* (L.) Osbeck	Lemon	Leaves	Use for scouring teeth	[115]
				Good as a mouth freshener	
	*Citrus medica* L.	Citron	Leaves and rind of fruits	Good for scouring teeth	[115]
	*Curcuma angustifolia* Roxb.	East Indian arrowroot	Rhizome	Appling on gums for pyorrhoea	[115]
	*Ficus hispida* L.f.	Hairy fig	Latex	Use for toothache	[115]
	*Ipomoea carnea* Jacq.	Bush morning glory	Leaves	Good for toothache	[115]
	*Jatropha curcas* L.	Physic nut	Twigs	Use as a toothbrush	[115]
				Good against dental caries	
	*Juglans regia* L.	Walnut	Bark and leaves	Use for scouring teeth	[115]
	*Mangifera indica* L.	Mango	Leaves	Use for scouring teeth	[115]
	*Murraya koenigii* (L.) Spreng.	Curry leaf	Stem	Use for scouring teeth and for healthy gums	[115]
	*Carya illinoinensis* (Wangenh.) K.Koch	Pecan	Leaves	Used for scouring teeth	[115]
				Good for gums	
	*Plumbago zeylanica* L.	Ceylon leadwort	Stem	Good for scouring teeth	[115]
	*Prunus cerasoides* Buch.-Ham. ex D.Don	Wild Himalayan cherry	Twigs	Use for scouring teeth	[115]
	*Psidium guajava* L.	Guava	Leaves and stem	Use for scouring teeth	[115]
	*Robinia pseudoacacia* L.	Black locust	Bark	Good for toothache	[115]
	*Vitex negundo* L.	Chinese chaste tree	Twigs	Good for cleaning teeth	[115]
				Good for pyorrhoea, gum inflammation, dental caries	
	*Zanthoxylum armatum* DC.	Winged prickly ash	Twigs	Use for scouring teeth	[115]

**Table 6:** Traditional usage of medicinal plants in cosmetic treatments of Sri Lanka.

No	Family	Scientific name	English name	Vernacular name	Traditional uses	Reference/s
1	Acanthaceae	*Barleria prionitis* L.^*∗*^	Crossandra	Katu karandu	The juice of the crushed leaves is applied to promote skin and scalp health	[116, 129]
					Prevent early grey or white hair	
2		*Justicia adhatoda* L.	Malabar nut	Adhatoda	Herbal toothpaste formulated with other plants is effective for oral hygiene	[116]
3	Acoraceae	*Acorus calamus* L.^*∗*^	Sweet flag	Wada-kaha	Ground rhizome is applied for pediculosis and to improve skin complexion	[116, 130]
4	Amaranthaceae	*Alternanthera sessilis* (L.) R.Br. ex DC.	Sessile joyweed	Mukunuwenna	Leaf juice promotes healthy hair and is used for manufacturing shampoos	[116, 130]
5	Amaryllidaceae	*Allium sativum* L.^*∗*^	Garlic	Sudulunu	Ground garlic mixed with honey or mixed with turmeric and honey is applied for curing pimples	[130]
6	Annonaceae	*Annona muricata* L.^*∗*^	Prickly custard apple	Katu-anoda	The juice of crushed young leaves is applied for pediculosis	[116, 129]
7	Apiaceae	*Centella asiatica* (L.) Urb.	Indian pennywort	Gotukola	The crushed leaves are used for damaged hair treatments and skin healing	[116, 131]
8	Apocynaceae	*Hemidesmus indicus *(L.) R. Br. ex Schult.	Indian sarsaparilla	Iramusu	The juice of the crushed leaves is applied to reduce hair loss and to improve skin complexion	[116, 129]
9		*Holarrhena pubescens* Wall. ex G.Don^*∗*^	Kurchi	Kelinda	The decoction of the bark is used as a mouth wash	[131]
10	Arecaceae	*Areca catechu* L.^*∗*^	Areca nut	Puwak	The decoction of the flowers is used as a mouth wash and is used in toothpaste manufacturing	[116, 131]
11		*Caryota urens* L.^*∗*^	Wine palm	Kithul	The bark and tender flowers improve hair growth	[116]
12		*Cocos nucifera* L.	King coconut/Pol	Thambili/Coconut	Kernel oil promotes hair growth	[116]
13	Asparagaceae	*Asparagus racemosus* Willd.	Wild asparagus	Hathawariya	The juice of the crushed leaves is applied to improve hair colour, promote hair growth, and prevent hair loss and early grey hair or white hair	[130]
					Possesses antiaging properties	
					The juice of the crushed leaves is applied to improve skin complexion and to remove skin freckles	
14	Asphodelaceae	*Aloe vera* (L.) Burm.f.	Aloe plant	Komarika	Sustaining youthful appearance, leaf gel is used to remove dead cells around the eye, freckles, and for curing pimples,	[116, 131]
					Leaf gel is applied to prevent hair loss and leaf gel with passion fruit (*Passiflora edulis* Sims) is applied to treat dandruff	
15	Asteraceae	*Eclipta prostrata* (L.) L.	False daisy	Keekirindiya	Leaf oil promotes hair growth	[116, 131]
					The juice of the crushed leaves is used to treat skin discolorations, and it possesses antiaging properties	
16		*Baccharoides anthelmintica* (L.) Moench^*∗*^	Purple fleabane	Sanninayam	Crushed seeds are applied to treat freckles	[132]
					Crushed seeds with lime juice is applied for pediculosis	
17	Berberidaceae	*Berberis aristata* DC.^*∗*^	Indian barberry	Daruharidra	The decoction of the bark is used as a mouth wash	[131]
18	Bombacaceae	*Bombax ceiba* L.^*∗*^	Red silk cotton tree	Imbul	Crushed thrones are applied to improve the skin complexion, to remove freckles, and for curing pimples	[129]
					Seed oil is used for manufacturing soaps	
19	Brassicaceae	*Brassica juncea* (L.) Czern.^*∗*^	Indian mustard	Aba	Seed oil is applied on the hands and legs to soften the skin and improve the complexion	[129]
20	Calophyllaceae	*Mesua ferrea* L.	Iron wood	Na	The crushed flower stamens mixed with khas-khas powder is applied on the body to remove the malodors of the body, remove freckles, and improve complexion by frequent application	[116]
21	Cannabaceae	*Celtis timorensis* Span.^*∗*^	Stinkwood	Gurenda/Burenda	Wood powder mixed with gingelly oil is applied on the skin for dryness to improve complexion	[116]
22	Caricaceae	*Carica papaya* L.	Papaya	Gaslabu	The ripe fruit is used to remove freckles	[116]
23	Celastraceae	*Kokoona zeylanica* Thwaites		Kokun	Soaps made from the crushed bark with water have been used for bathing by men and women since ancient times	[132]
					The bark powder is used to treat pimples and to improve skin complexion	
24	Clusiaceae	*Garcinia × mangostana* L.^*∗*^	Mangosteen	Mangus	The decoction of the bark is used as a mouth wash for healthy gums and teeth	[116]
25		*Garcinia quaesita* Pierre	Red mango	Goraka	It is used for manufacturing toothpastes	[131]
26	Combretaceae	*Terminalia arjuna* (Roxb. ex DC.) Wight & Arn.^*∗*^	Arjuna myrobalan	Kumbuk	The bark powder mixed with honey is applied on pimples, and the bark is used for manufacturing toothpaste	[131]
27		*Terminalia bellirica* (Gaertn.) Roxb.	Beleric myrobalan	Bulu	Applying the seeds' oil improves the black colour of hair and controls hair loss	[131]
28		*Terminalia chebula* Retz.	Myrabalans	Aralu	The infusion of the powdered, dried, young fruit is used as a mouthwash	[116, 129]
					Possesses antiaging properties and is used to manufacture yellow colour dye	
29	Convolvulaceae	*Argyreia populifolia* Choisy^*∗*^	Sri Lankan elephant creeper	Giritilla	The juice of the young shoots is applied on the gums for sanitation	[116]
30		*Cuscuta chinensis* Lam.^*∗*^	Dodder	Agamula nathi wal	Concentrated plant extract with gingelly oil upon boiling is applied to improve the black color of hair and prevent hair loss, and it is used to treat dandruff	[132]
31		*Evolvulus alsinoides *(L.)L.^*∗*^	Slender dwarf morning- glory	Vishnukranthi	Plant extracted oil is applied to promote healthy hair growth	[130]
32		*Ipomoea pes-caprae* (L.) R. Br.^*∗*^	Goats foot creeper	Binthamburu	The decoction of the leaves is used for foot health	[131]
33	Costaceae	*Cheilocostus speciosus* (J.Koenig) C.D.Specht^*∗*^	Crape ginger	Thebu	The bark is used to remove freckles, especially those that occurred during chicken pox	[132]
34	Cucurbitaceae	*Cucumis melo* L.^*∗*^	Bitter cucumber	Gon kakiri	The crushed fruit mixed with the nux-vomica tree (*Strychnos nux-vomica* L.) seeds is used to treat pimples	[131]
35		*Cucumis sativus* L.	Cucumber	Pipingna	Fruit slices are used to treat darkness under the eyes and are used for skin cooling, soothing, and healing	[116]
36		*Trichosanthes cucumerina* L.^*∗*^	Wild snake gourd	Dummalla	The decoction of leaves is used to control early grey or white hair	[131]
37	Cyperaceae	*Cyperus rotundus* L.	Nutgrass	Kalanduru	Crushed tubers are used to treat acne	[129]
38	Dilleniaceae	*Dillenia retusa* Thunb.		Godapara	The fruit pulp is used to cleanse the scalp and promote healthy hair growth	[116, 131]
39	Ebenaceae	*Diospyros malabarica* (Desr.) Kostel.^*∗*^	Riber ebony	Timbiri	The decoction of unripe fruit slices is used as a mouthwash or gargle for mouth sanitation	[116]
40	Elaeocarpaceae	*Elaeocarpus serratus* L.	Wild olive	Weralu	The leaves are boiled with true lime slices to clean the scalp and hair	[116]
41	Euphorbiaceae	*Jatropha curcas* L.	Purging nut	Rata endaru	The decoction of the bark is used for mouth sanitation	[116]
42	Fabaceae	*Abrus precatorius* L.^*∗*^	Wild liquorice	Olinda	Grounded seeds with true lime are used to treat acne	[116]
43		*Caesalpinia bonduc* (L.) Roxb.^*∗*^	Molucca bean	Kumburu	Seeds' oil is applied to cure pimples, and fried seeds' powder is used for brushing the teeth	[131]
44		*Crotalaria verrucosa* L.^*∗*^	Blue rattle weed	Nil-adanahiriya	Gargling and the infusion of young shoots and leaves promote mouth sanitation	[116]
45		*Entada rheedii* Spreng.^*∗*^	Elephant creepes mackay	Pus well	Seed kernel powder mixed with white sandalwood powder in true lime juice is used to treat pimples	[116, 132]
					The ground seeds, stem, and bark, along with water, are used to clean the scalp and hair	
46		*Indigofera tinctoria* L.	Indigo	Nilawari	Medicated oil prepared with fresh leaf juice and king coconut oil is applied to improve skin complexion	[116]
					Ground with false daisy, turmeric, and kurchi, it is applied to control hair loss, and the oil extracted from the leaves promotes hair growth	
47		*Mimosa pudica* L.^*∗*^	Sensitive plant	Nidikumba	The ground aerial parts with gingelly oil are applied on the skin because of healing properties, and a decoction of the roots is used as a mouth wash to improve mouth hygiene	[116]
48		*Pongamia pinnata* (L.) Pierre	Indian beech	Karanda	Seed oil is used in soap manufacturing because of its antimicrobial properties	[130]
49		*Pterocarpus marsupium* Roxb.^*∗*^	Indian kino tree	Gammalu	Dissolved dried gum in warm water is used as a mouthwash for healthy gums and teeth	[116, 131]
50		*Pterocarpus santalinus* L.f.	Red sandalwood	Rath-handun	Crushed heartwood mixed with turmeric powder and milk is used to treat pimples and is used in soap manufacturing	[116, 130]
51		*Senna alata* (L.) Roxb.	Candle bush	Eththora	The leaves are used in antiseptic soap manufacturing because of antimicrobial properties	[116, 129]
52		*Senna auriculata* (L.) Roxb.^*∗*^	Tanner's cassia	Ranawara	Crushed flowers are applied to the skin to improve complexion	[130]
53		*Tephrosia purpurea* (L.) Pers.	Purple tephrosia	Kathurupila	The roots are used for mouth sanitation and are used in manufacturing toothpastes	[116, 131]
54		*Trigonella foenum-graecum* L.	Fenugreek	Uluhal	Boiled with unripe true limes to treat dandruff	[131]
55	Hypoxidaceae	*Curculigo orchioides* Gaertn.^*∗*^	Black musale	Binthal	The crushed tuber has been used to improve the beauty of the face by men and women since ancient times	[131]
56	Lamiaceae	*Ocimum tenuiflorum* L.	Holy basil	Heen maduruthala	The extracted leaf oil is used as a fragrant agent, and the juice of the crushed leaves is used to treat pimples and freckles	[130]
57		*Pogostemon heyneanus* Benth.^*∗*^	Patchouli	Gas-kollankola	Infusion of fresh or dried leaves is used as a mouthwash for healthy gums and teeth	[116]
58		*Premna obtusifolia* R.Br.^*∗*^		Heen midi	Leaves mixed with coconut oil are used to clean the scalp and hair	[130]
59		*Tectona grandis* L.f.^*∗*^	Teak	Thekka	Dried fruit powder promotes hair growth	[116]
60	Lauraceae	*Cinnamomum verum* J.Presl	Ceylon cinnamon	Kurundu	The dried inner bark is used as a mouth wash for gum and teeth sanitation	[116]
					Cinnamon powder mixed with honey is applied to treat pimples	
61	Lecythidaceae	*Barringtonia acutangula* (L.) Gaertn.^*∗*^	Indian oak	Midella	The decoction of the leaf, bark, and flower is used as a mouth wash for healthy, strong gums	[130]
62		*Careya arborea* Rroxb.^*∗*^	Patana oak	Kahata	Bark gum is used to soften the skin	[132]
63		*Couroupita guianensis* Aubl.^*∗*^	Cannon ball tree	Sal	Leaves stimulate the dermal fibroblast proliferation	[116]
64	Lythraceae	*Lawsonia inermis* L.	Henna	Marathondi	Leaves are boiled in water with true lime fruit pieces are used to treat grey or white hair, and the leaves stimulate hair growth and are used to colour nails	[116]
65		*Punica granatum* L.	Pomegranate	Delum	The decoction of the roots and fruit rind is used as a mouth wash, and the decoction of the leaves is good for the eyes	[116, 131]
66	Malvaceae	*Abutilon indicum* (L.) Sweet^*∗*^	Country mallow	Behethanoda	Crushed leaves are used as a mouth wash	[131]
67		*Gossypium arboreum* L.^*∗*^	Cotton	Kapu	Leaves stimulate dermal fibroblast proliferation	[116]
68		*Abelmoschus moschatus* Medik.	Musk mallow	Kapukinissa	Seeds' paste is applied for skin itching, and chewing the seeds removes the malodors of the mouth	[116, 129]
69		*Hibiscus rosa-sinensis* L.	Shoe flower	Pokuru wada	Oil prepared by boiling the leaves and flowers with coconut oil or gingelly oil is applied to promote healthy hair growth, prevent early grey or white hair, hair loss, and dandruff	[116]
					Crushed flowers and leaves are applied to remove suntan, to cure pimples, and to remove freckles on the skin	
70		*Theobroma cacao* L.	Coco	Kokova	Possesses emollient properties and is used to soften and treat dry skin and chapped lips	[116]
71	Meliaceae	*Azadirachta indica* A.Juss.	Neem	Kohomba	Crushed leaves mixed with true lime juice are applied on pimples, and a decoction of the roots is used as a mouth wash and is used in manufacturing soaps and toothpastes	[131]
72	Menispermaceae	*Coscinium fenestratum* (Goetgh.) Colebr.	Calumba wood	Weniwel	Stem powder mixed with honey is used to exfoliate the skin, and it possess antiacne properties	[116, 130]
73	Moraceae	*Ficus racemosa* L.	Country fig	Attikka	Crushed leaves are used to treat freckles and skin discoloration	[129]
74	Myristicaceae	*Myristica fragrans* Houtt.	Nutmeg	Sadhikka	The seed extracted oil is used in soap and toothpaste manufacturing, the herbal shampoo is prepared for pediculosis, it is used to clean the scalp and hair, and it is used to protect skin keratinocytes from UV B- induced damage	[116, 130]
75	Myrtaceae	*Melaleuca leucadendra* (L.) L.	Cajuput tree	Lothsumbulu	Improves skin complexion, and the bark acts as a stimulant and tonic	[116]
76		*Syzygium aromaticum* (L.) Merr. & L.M.Perry	Clove	Karabu	It is used in toothpaste manufacturing	[116, 129]
77		*Syzygium cumini* (L.) Skeels^*∗*^	Indian black berry	Madan	The bark is used to prepare mouth washes for healthy gums and teeth used in manufacturing toothpastes	[116, 130]
78	Nymphaeaceae	*Nymphaea nouchali* Burm.f.	Blue water lily	Nil manel	The flowers are mixed with cow milk and stored in a covered clay pot approximately a month to colour grey or white hair into black	[130]
79	Oleaceae	*Jasminum grandiflorum* L.	Jasmine	Samanpichcha	Ground roots and flowers improve skin discoloration, used to treat skin discolorations, plant-extracted oil is applied to cool the scalp	[116, 130]
80		*Jasminum multiflorum* (Burm.f.) Andrews^*∗*^	Sambac Jasmine	Geta pichcha	Paste made by grinding the flower buds is applied on the skin for improved complexion	[116]
81	Oxalidaceae	*Averrhoa bilimbi* L.^*∗*^	Bilimbi	Bilin	Leaf juice is applied to cure pimples, herbal shampoo prepared from the plant is effective for pediculosis	[116]
82	Pedaliaceae	*Sesamum indicum* L.	Gingelly	Thel-thala	Liniments to promote hair growth, possesses healing properties	[116]
83	Phyllanthaceae	*Phyllanthus emblica* L.	Emblic myrobalan	Nelli	Improves skin complexion, prevents hair loss and early grey or white hair	[131]
					Used in manufacturing herbal hair oil, shampoo, conditioner, and skin care products	
84	Plantaginaceae	*Bacopa monnieri* (L.) Wettst.	Thyme leaved gratiola	Lunuwila	Possesses antiaging properties and antidandruff properties	[116]
85	Poaceae	*Cymbopogon citratus* (DC.) Stapf	Lemon grass	Sera	Shampoo containing essential oil is effective for pediculosis and dandruff	[116, 130]
86		*Chrysopogon zizanioides* (L.) Roberty	Khas-khas	Sawandara	Root-extracted oil is used in the perfume industry and soap manufacturing and is used as a fragrant agent, and the crushed roots are applied on the skin	[116, 130]
87	Ponterderiaceae	*Monochoria vaginalis* (Burm.f.) C.Presl	Oval leafed pondweed	Diyahabarala	Root infusion is used as a mouthwash for mouth inflammations	[116]
88	Rubiaceae	*Morinda citrifolia* L.^*∗*^	Indian mulberry	Ahu	The juice of the fruits in salt water is used as a mouthwash for healthy gums, and the crushed leaves are used to exfoliate the skin and for dermal fibroblast proliferation	[116, 129]
89		*Rubia cordifolia* L.	Heart leaved madder	Velmadata	Crushed roots mixed with honey are used to remove freckles and skin discolorations	[130]
90	Rutaceae	*Acronychia pedunculata* (L.) Miq.	Claw flowered laurel	Ankenda	Crushed leaves are used to treat pimples	[116]
91		*Aegle marmelos* (L.) Corrêa	Bael fruit tree	Beli	Ripe fruit mixed with green gram powder is applied to improve skin complexion and can be used as an eye lotion	[116, 131]
92		*Citrus aurantiifolia* (Christm.) Swingle	True lime	Dehi	Acts as a cleanser, unripe fruits are boiled with fenugreek to treat dandruff, fruits are ground with lotus leaves to treat pediculosis	[131]
93		*Citrus hystrix* DC.^*∗*^	Kaffir lime	Gada dehi	Fruit juice is used to treat dandruff and is used in aromatic baths and as a shampoo	[116]
94		*Melicope lunu-ankenda* (Gaertn.) T.G. Hartley^*∗*^		Lunu-ankenda	Crushed leaves are used to improve complexion	[116]
95		*Murraya koenigii* (L.) Spreng.^*∗*^	Curry leaf	Karapincha	The juice of the crushed leaves is used to promote healthy hair growth	[129]
96		*Ruta graveolens* L.	Garden rue	Aruda	Herbal oil prepared with fresh leaves and pure coconut oil is applied for healthy hair	[129]
97	Santalaceae	*Santalum album* L.	Sandalwood	Sudu handun	Heartwood powder mixed with lime juice is applied gently on pimples to improve skin complexion, the powder mixed with cow milk is used to treat freckles, and it possesses antiaging properties	[116, 130]
98	Sapindaceae	*Schleichera oleosa* (Lour.) Merr.	Ceylon oak	Kon	Seed oil is applied to promote healthy hair	[116]
99	Sapotaceae	*Mimusops elengi* L.	Bullet wood tree	Munamal	The decoction of the mature bark is used as a mouthwash for healthy gums and teeth, herbal toothpaste formulated with other plants is effective for oral hygiene	[116, 130]
100	Solanaceae	*Datura metel* L.^*∗*^	Datura	Attana	The root powder is applied on the gums for mouth sanitation	[116]
101	Symplocaceae	*Symplocos cochinchinensis* (Lour.) S. Moore^*∗*^		Bombu	Bark decoction is used as a mouthwash for healthy gums	[116]
102	Theaceae	*Camellia sinensis* (L.) Kuntze	Tea	Thae	Possesses antiwrinkle properties	[116]
103	Vitaceae	*Leea indica* (Burm. f.) Merr.^*∗*^	Bandicoot berry	Gurulla	Crushed leaves are applied on skin patches to improve skin complexion	[130]
104	Zingiberacea	*Alpinia malaccensis* (Burm.f.) Roscoe		Rankihiriya	Flower bud juice in lukewarm water is used as a mouth wash for healthy gums and teeth	[116]
105		*Curcuma longa* L.	Turmeric	Ath kaha	The crushed rhizome is used to improve skin complexion, prevents UV B-induced skin aging	[116]
106		*Curcuma zedoaria* (Christm.) Roscoe	Zedoary	Haran kaha	Crushed rhizome improves skin complexion, and it is used as a fragrant agent	[130]
107		*Kaempferia galanga* L.^*∗*^	Java galanga	Ingurupiyali	Dried sliced rhizome infusion is used as a mouth wash, powdered rhizome with coconut oil on the skin to improves skin complexion, improves lustrous of hair, possesses antidandruff properties and skin whitening properties	[116, 129]
108		*Zingiber officinale* Roscoe	Ginger	Inguru	Juice-extracted ginger pulp is applied on pimples, and it is used to improve skin complexion	[129]

^*∗*^The plant species that are currently not used in the herbal cosmetic industry of Sri Lanka (in comparison with the survey conducted on “Emerging herbal cosmetic production in Sri Lanka: Identifying the possible interventions for the development of herbal cosmetic industry” [12]).

## Data Availability

The data used to support the findings of this study are included in the article.
